# The Relationship between Renal Dysfunction and Abnormalities of the Immune System in Patients with Decompensated Cirrhosis

**DOI:** 10.5402/2012/123826

**Published:** 2012-12-26

**Authors:** Eiji Kakazu, Yasuteru Kondo, Tooru Shimosegawa

**Affiliations:** Division of Gastroenterology, Tohoku University Hospital, 1-1 Seiryo, Aobaku, Sendai 980-8574, Japan

## Abstract

In patients with advanced cirrhosis, not only hepatocellular carcinoma but also bacterial infections, such as spontaneous bacterial peritonitis (SBP) or pneumonia, are frequent clinical complications in such immune-compromised patients. These pathologies often progress to renal dysfunction, especially hepatorenal syndrome (HRS). The central pathology of HRS is splanchnic arterial vasodilation and hyperpermeability followed by bacterial translocation (BT). BT induces a severe inflammatory response in the peritoneal lymphoid tissue, with the activation of the immune systems and the long-lasting production of vasoactive mediators that can impair the circulatory function and cause renal failure. Recent studies report that the plasma amino acid imbalance appeared to be related to an abnormality of the immune system in patients with decompensated cirrhosis. This paper can provide a new approach for future studies of the pathology in cirrhotic patients with renal dysfunction.

## 1. Introduction

Various complications occur in patients with decompensated cirrhosis. Renal dysfunction, a parameter included in the MELD score [1, 2], is the most important prognostic factor. Some kinds of renal dysfunction appear in patients with decompensated cirrhosis (Table 1), and it is necessary to treat the pathogenesis adequately. Gastrointestinal bleeding, overload of diuretic drugs, and repeated drainage of ascites induce hypovolemia and, frequently, hepatorenal syndrome (HRS). P. Ginés and V. Arroyo proposed diagnostic criteria for HRS [3, 4], which is now used worldwide (Table 2). HRS, which is the main cause of the renal dysfunction in decompensated cirrhosis, has not been completely elucidated [4–6]. There are two types of HRS. Type-2 HRS is characterised by moderate renal failure (serum creatinine from 1.5 to 2.5 mg/dl), with a steady or slowly progressive course, and Type-1 HRS is characterised by a rapid progressive renal failure defined by the doubling of the initial serum creatinine concentrations to a level greater than 2.5 mg/dl in less than 2 weeks. The natural prognosis of type-1 HRS is very poor [7]. The central pathology of HRS is splanchnic arterial vasodilation and hyperpermeability followed by BT, which easily occurs in decompensated cirrhosis. On the other hand, in patients with advanced cirrhosis, various metabolic disorders involving glucose, amino acids (AAs), lipids, vitamins, and minerals appear. It was recently reported that the plasma amino acid imbalance appeared to be related to an abnormality of the immune system in patients with decompensated cirrhosis [8–10]. In this paper we will discuss the causes of HRS based on previous reports.

## 2. Hepatorenal Syndrome (HRS) and Renal Autoregulation System

Portal hypertension occurs, followed by intrahepatic vascular resistance, which is the progression of hepatic fibrosis in patients with cirrhosis. Furthermore, the effective circulating blood volume decreases and the extracellular fluid volume increases because of the splanchnic arterial vasodilation and hyperpermeability, followed by portal hypertension. On the other hand, the renal blood flow is compensated in patients with early cirrhosis, because the autoregulation system maintains the renal blood flow, even if the renal artery pressure fluctuates between 80 and 180 mmHg.

### 2.1. Rennin-Angiotensin-Aldosterone System (RAAS)

RAAS is the central hormonal regulation that controls the kidney bloodstream. Increasing angiotensin II promotes the reabsorption of sodium by distal renal tubules and collecting kidney tubules and maintains the glomerular filtration rate (GFR) by the contraction of the efferent arterioles. In patients with cirrhosis, sodium retention occurs in response to lower body negative pressure, which was associated with increased RAAS activity [9]. A previous study reports that the RAAS is activated in 50–80% of patients with decompensated cirrhosis and HRS accelerates the RAAS [10]. Another study reported that RAAS is activated by diuretic drugs [11]. 

### 2.2. Vasopressin

Vasopressin, which is the main antidiuretic hormone, is synthesized by the hypothalamus and stored in nerve endings of the posterior pituitary gland. The secretion is usually promoted by an increase of the plasma osmolarity or the decrease of the blood volume, but even when the plasma osmolality falls, the production continues to be promoted in patients with HRS [12, 13]. There are three vasopressin receptors [14]: V1a, V1b, and V2. The V2 receptor in collecting kidney tubules promotes the reabsorption of water and decreases the urine output. 

### 2.3. Sympathetic Nervous System

**Figure 1:**
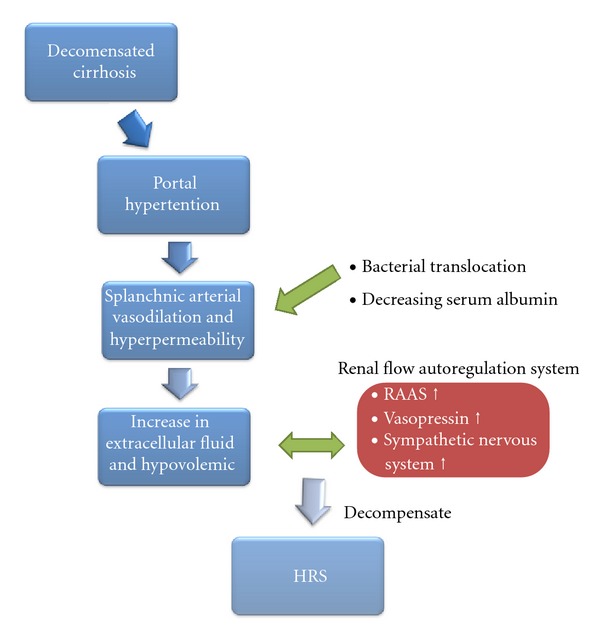
Pathology of hepatorenal syndrome.

An efferent pathway of the sympathetic nervous system to the kidney reaches the juxtaglomerular apparatus, renal tubular, and blood vessel floor, and when a renal sympathetic nerve centrifugal is stimulated, renin secretion is promoted [15], but the renal artery shrinks through *α* 1A receptor and the renal blood volume decreases. Sympathetic nervous activity is enhanced in patients with cirrhosis. In patients with HRS, the sympathetic nerve is activated [16–18] and GFR is decreased by the contraction of an afferent arteriole in the glomerulus, and the reabsorption of sodium by renal tubules is promoted. Furthermore, the renal blood volume is also maintained by the intricate dynamics of the glomerulotubular balance [19], tubuloglomerular feedback [20], and myogenic response [21]. But when such a state continues for an extended period, the dynamics of renal compensation fail to recover the renal blood volume leading to HRS (Figure 1). Although increasing angiotensin II and the contraction of an efferent arterioles maintain GFR, the effective circulation blood volume cannot recover from hypovolemia because of the increased extracellular fluid caused by the reabsorption of water and Na^+^. Finally, through such a vicious circle the patients with decompensated cirrhosis develop edema, ascites, low cardiac output, and HRS. A synthetic decline of the albumin, which plays a central role in the maintenance of the plasma osmolality, is also one of the important causes. Indeed, it was demonstrated by a randomized controlled trial that the administration of albumin prevents renal dysfunction in patients with spontaneous bacterial peritonitis (SBP) [22], and it is a basic treatment in patients with decompensated cirrhosis. Furthermore, it was reported that, terlipressin, a drug that causes blood vessel shrinkage, is effective for HRS and more effective in combination with albumin, although its effect is only about 30–50% [23–26]. Other vasoconstrictor drugs have been found to be inadequate [27–29].

## 3. Bacterial Translocation (BT) and Immune Abnormality in Patient with Cirrhosis

**Figure 2:**
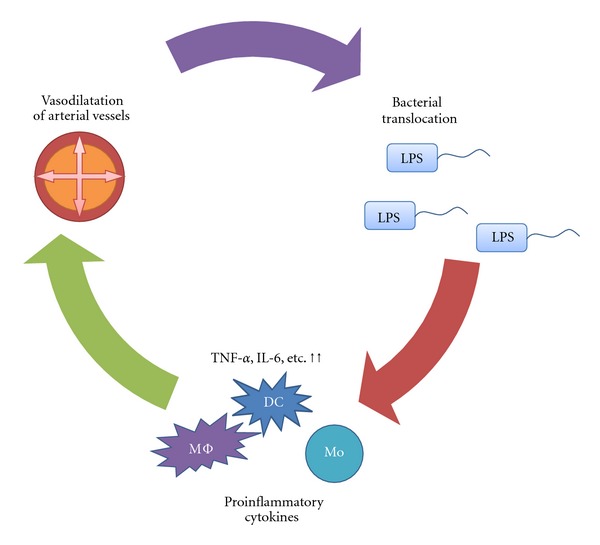
Mechanism of the splanchnic arterial vasodilation and hyperpermeability.

Bacteria can normally be detected in underlying intestinal tissue without associated injury because organisms are usually efficiently removed by phagocytes. However, bacterial translocation (BT) is the migration of bacteria or bacterial products from the intestinal lumen to mesenteric lymph nodes [30, 31]. BT is deeply related to the splanchnic arterial vasodilation and hyperpermeability (Figure 2). In the normal gut mucosa, monocytes and particularly DCs are in charge of providing innate protection against microorganisms. A bacterial stimulus from the intestinal tract activates antigen presentation cells (monocytes, macrophages, and dendritic cells (DCs)), and these cells produce proinflammatory cytokines (TNF-alpha and IL-6 et al) and substances that cause vasodilation (NO, bories et al.) [32]. It is well known that the levels of many proinflammatory cytokines (TNF-alpha, IL-6, IL-1*β*, etc.) are higher in the plasma of patients with cirrhosis than in that of healthy subjects [33, 34]. Previous studies using a cirrhotic mouse model proved the existence of BT by detecting bacterial DNA in a mesentery lymph node, the plasma, and ascites, and the BT continued to promote the active status of immune cells [35–38]. Furthermore, the prevalence of BT significantly increased according to the Child-Pugh classification: 3.4% in Child A, 8.1% in Child B, and 30.8% in Child C patients [39]. Although it is unclear why BT easily occurs in decompensated cirrhosis, three primary mechanisms promote BT from the gastrointestinal tract: intestinal bacterial over growth [40–42], increased intestinal permeability [43, 44], and immune abnormality. These mechanisms can act in concert to promote synergistically translocation.

## 4. Amino Acid Imbalance and Immune Abnormalities in Patients with Cirrhosis

**Figure 3:**
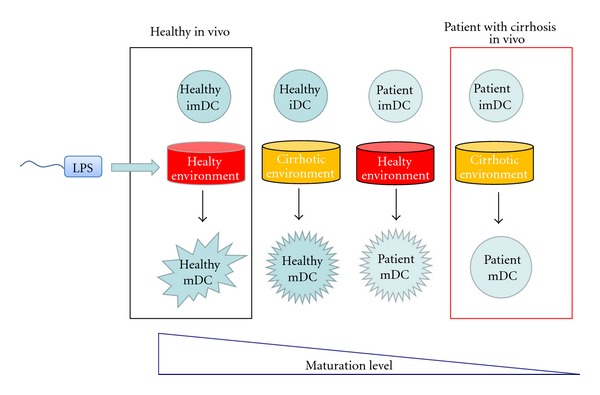
Dysfunction of dendritic cells in patient with cirrhosis.

For immune abnormalities in patients with advanced cirrhosis, previous studies have described the dysfunction of immune cells, especially DCs [45–48], and our study demonstrated that, in advanced cirrhosis, the extracellular amino acid environment also tends to impair the maturation of DCs [49] (Figure 3). Concerning the mechanism that underlies this phenomena, the amino acid imbalance in the plasma of patients with advanced cirrhosis influenced the mTOR/S6K signaling pathway of the DCs [49].

**Figure 4:**
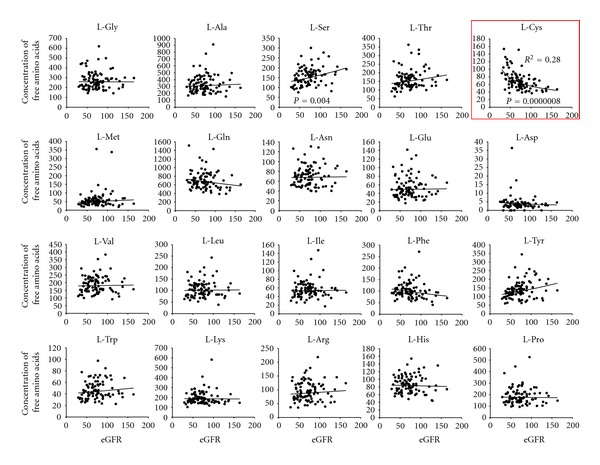
Free amino acids related to renal function in patients with advanced cirrhosis eGFR are calculated by [8].

**Figure 5:**
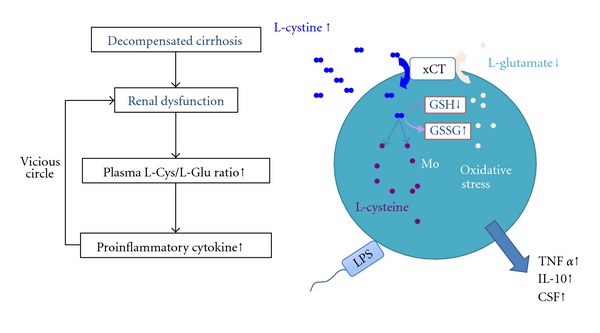
The amino acid imbalances influence the function of monocytes in cirrhotic patients with renal dysfunction.

Furthermore, branched-chain amino acids (BCAAs) enhance the maturation and function of myeloid DCs ex vivo in patients with advanced cirrhosis [49]. On the other hand, we revealed that the free amino acid concentration L-Cystine (L-Cys) correlated inversely with the glomerulus filtration rate (eGFR) in patients with cirrhosis (Figure 4), and high levels of L-Cys increase the production of TNF-alpha from monocytes [50]. Concerning the mechanism that underlies this phenomena, high extracellular levels of L-Cys enhanced the exchange L-Cys/L-Glu antiport of monocytes via xCT and decreased the intracellular GSH/GSSG ratio under the amino acid condition of advanced cirrhosis (Figure 5). Furthermore, we reported that the mRNA expression of TNF-alpha and xCT were significantly higher in monocytes of patients with decompensated cirrhosis than in those of healthy volunteer [50]. Recently, it has become clear that AAs are not only important as substrates for various metabolic pathways, but also activate a nutrient-sensitive signaling pathway in synergy with insulin [51–53], and that extracellular AAs influence the function of immune cells [54–57]. The amino acid imbalance is considered one of the reasons that the immune cells cannot normally exclude bacteria and so the inflammation continuous may relate to the development of HRS in patient with advanced cirrhosis. 

## 5. Evaluation of Renal Dysfunction in Decompensated Cirrhosis

Although serum creatinine is commonly used to evaluate renal function, it does not exactly reflect GFR in patients with cirrhosis [58]. Because the value of serum creatinine varies depending on the amount of skeletal muscle, the GFR is overestimated in patients with cirrhosis who have decreased amounts of skeletal muscle [59, 60]. Of course, the creatinine clearance is the same. On the other hand, the inulin clearance [61], which is the global standard measurement for GFR, can reflect GFR correctly, but repeat measurement is difficult clinically because the method is very complicated. Recently, it, using cystatin C, one of the serum proteins, was effective for evaluating the renal function of patients with cirrhosis [62, 63]. It is a potent inhibitor of lysosomal proteinases and one of the most important extracellular inhibitors of cysteine proteases. It is produced by nucleated cells of the whole body and acts as a cysteine protease inhibitor in the living body. Cystatin C in the blood is filtered by renal glomeruli and is reabsorbed by proximal renal tubules [64]. It is not influenced by the creatinine level or the amount of skeletal muscle. Although serum cystatin C determination could be a valuable tool in patients with cirrhosis for early diagnosis of moderately impaired renal function [65], further investigation is needed to clarify its effectiveness, for evaluating patients with decompensated cirrhosis. 

## 6. Summary

HRS is one of the most severe complications in patients with decompensated cirrhosis. Although liver transplantation is the only curative treatment for HRS, renal failure is a risk factor for a poor outcome of liver transplantation. Further investigation of the pathology and therapy of HRS is needed.

## Figures and Tables

**Table 1:** The pathology of renal dysfunction in patients with decompensated cirrhosis.

	Pathology
HRS	HRS is classified into two types: type 1 is characterized by a doubling of the serum creatinine level to more than 2.5 mg/dL in less than 2 weeks; type 2 is characterized by a stable or less rapidly progressive course than in type 1.

Hypovolemia-induced renal failure	Renal flow losses because of excessive diuretic therapy or gastrointestinal losses as a result of diarrhea from excessive lactulose administration or gastrointestinal infection. Renal failure occurs soon after the onset of hypovolemia.

Parenchymal renal disease	Acute or chronic parenchymal renal disease should be suspected as a cause of renal failure when proteinuria, hematuria, or both are present and ideally should be confirmed by renal biopsy

Drug-induced renal failure	Nonsteroidal anti-inflammatory drugs or antibiotics suggest drug-induced renal failure.

**Table 2:** Diagnostic criteria of hepatorenal syndrome (HRS).

Major criteria	
(i) Low glomerular filtration rate, as indicated by serum creatinine level greater than 1.5 mg/dL or 24-hour creatinine clearance lower than 40 mL/minute	
(ii) Absence of shock, ongoing bacterial infection, fluid loss, and current treatment with nephrotoxic drugs	
(iii) No sustained improvement in renal function (decrease in serum creatinine to 1.5 mg/dL or less or increase in creatinine clearance to 40 mL/minute or more) following the diuretic withdrawal and expansion of plasma volume with 1.5 L of a plasma expander	
(iv) Proteinuria lower than 500 mg/day and no ultrasonographic evidence of obstructive uropathy or parenchymal renal disease	
Additional criteria	
Urine volume lower than 500 mL/day	
Urine sodium lower than 10 mEq/L	
Urine osmolality greater than plasma osmolality	
Urine red blood cells less than 50 per high-power field	
Serum sodium concentration lower than 130 mEq/L	

All major criteria must be present for the diagnosis of hepatorenal syndrome. Additional criteria are not necessary for the diagnosis, but provide supportive evidence.
